# Novel Metal Nanomaterials and Their Catalytic Applications

**DOI:** 10.3390/molecules200917070

**Published:** 2015-09-17

**Authors:** Jiaqing Wang, Hongwei Gu

**Affiliations:** Key Laboratory of Organic Synthesis of Jiangsu Province, College of Chemistry, Chemical Engineering and Materials Science & Collaborative Innovation Center of Suzhou Nano Science and Technology, Soochow University, Suzhou 215123, China; E-Mail: kinglion1@163.com

**Keywords:** nanomaterial, heterogeneous catalyst, organic reaction, electro-catalysis, green chemistry

## Abstract

In the rapidly developing areas of nanotechnology, nano-scale materials as heterogeneous catalysts in the synthesis of organic molecules have gotten more and more attention. In this review, we will summarize the synthesis of several new types of noble metal nanostructures (FePt@Cu nanowires, Pt@Fe_2_O_3_ nanowires and bimetallic Pt@Ir nanocomplexes; Pt-Au heterostructures, Au-Pt bimetallic nanocomplexes and Pt/Pd bimetallic nanodendrites; Au nanowires, CuO@Ag nanowires and a series of Pd nanocatalysts) and their new catalytic applications in our group, to establish heterogeneous catalytic system in “green” environments. Further study shows that these materials have a higher catalytic activity and selectivity than previously reported nanocrystal catalysts in organic reactions, or show a superior electro-catalytic activity for the oxidation of methanol. The whole process might have a great impact to resolve the energy crisis and the environmental crisis that were caused by traditional chemical engineering. Furthermore, we hope that this article will provide a reference point for the noble metal nanomaterials’ development that leads to new opportunities in nanocatalysis.

## 1. Introduction

Catalysis is the driving force behind the development of the chemical industry, which can not only make us use natural resources more efficiently, but also reduce the pollution in the processes of the chemical industry. In the last century, catalysis has become the foundation of the large-scale production of the chemical and petroleum industry [1,2,3,4]. However, there still exist some problems to be solved [5,6]. With the development of nanotechnology, catalysis has ushered in some new challenges and opportunities.

In recent years, certain achievements have been made in the metal nanomaterials as heterogeneous catalysts [7,8,9,10]. These catalysts have a very high catalytic activity and selectivity for specific reactions. Nanocatalysts for catalytic chemical reactions mainly include the oxidation reaction [7], the reduction reaction [8], coupling reaction [9,10] and the electrochemical reaction [11,12,13,14,15]. They have attracted more and more workers’ attention. The main reason why nanomaterials have attracted so much attention is that they are a bridge between atoms and bulk materials. In addition, they have some special properties, such as the surface and interface effect (unusual properties of extremely small crystals that arise from the damage of a boundary between a material and its surrounding environment), small size effect (novel properties of extremely small crystals that arise from the decrease of the atom’s density of amorphous nanoparticles near the surface layer), quantum size effect (unusual properties of extremely small crystals that arise from confinement of electrons to small regions of space in one, two, or three dimensions) and macroscopic quantum tunnel effect (when the total energy is less than the barrier height, extremely small crystals can still pass through the barrier.), as well as, some potential applications, such as catalysis, biology, medicine and so on. This review article reviews noble metal nanomaterials as heterogonous catalysts applied into organic reactions and electro-catalysis in our group recently, which intends to combine nanomaterial preparation and organic chemistry or fuel cell systems to find new catalysts for low-cost, highly-selective chemical synthesis that is clean and energy-efficient. In catalytic organic reactions, the transformation of functional groups based on novel nanomaterials, can not only achieve “atom economy”, the chemical processes showed low energy consumption and induced low pollution at the same time. In electro-catalysis, the synthesized nanomaterials can enhance electrocatalytic activity for direct oxidation of methanol and formic acid, as well as, meet the demand for clean and energy-efficient fuel cell systems.

Therefore, we arrange the content of this review in the following way. Firstly, we introduce several platinum (Pt)-based nanocomplexes’ synthesis including FePt@Cu nanowires (NWs), Pt@Fe_2_O_3_ NWs and Pt@Ir zigzag bimetallic nanocomplexes. The former two kinds of novel NWs were used as “non-support” heterogeneous catalysts in oxidation, while the latter one exhibited good performance in reduction. Secondly, the facile synthesis of hybrid nanostructures (such as necklace-like Pt-Au nanostructures, three-dimensional Pt/Pd bimetallic nanodendrites and Au-Pt bimetallic nanocomplexes) is reviewed to demonstrate their high stabilities as electrocatalysts for the oxygen-reduction reaction. Thirdly, we discuss the synthesis of several other noble metal nanomaterials including Au, Pd and Ag, but also their excellent catalytic abilities. Lastly, we attempt to point out a novel solution of “green chemistry” based on heterogeneous nanocatalysts, in order to stimulate the future development of nanomaterials that ultimately contributes to addressing the current challenges and that leads to new opportunities in nanomaterials and nanocatalysis.

## 2. Pt Nanocomplexes and Catalytic Applications in Organic Reactions

Due to the quite high chemical stability and catalytic activity, Pt nanomaterials have been widely used in many fields, especially in catalysis. The chemical properties of Pt are inactive and stable in air and moisture. There existing partially-full *d* orbit in the outer layer of Pt results in it being easy to form complexes and some intermediates with high activity. Consequently, Pt is one of the most significant catalytic materials. Early in 1831, as catalysts, Pt had been successfully applied to the synthesis of sulfuric acid. From then on, Pt based catalysts have attracted more and more researchers because of their excellent catalytic activity, selectivity and stability. They play an important role in a wide field of medicine, environmental protection, energy, petrochemicals and fine chemicals. In 2009, Grimes and coworkers investigated nitrogen-doped titania nanotube arrays which were loaded with both Cu and Pt nanoparticles and successfully used the catalysts for high-rate solar photocatalytic conversion of CO_2_ and water vapor to hydrocarbon fuels [16]. Pt nanocatalysts for the reduction of *p*-nitrophenol have been also reported much more, such as the Pt nanoparticles decorated on reduced graphene oxide by the simultaneous reduction of graphene oxide and the metal ions in Mg/acid medium [17], dendrimer-templated and reverse microemulsion Pt nanoparticles [18] and reduced graphene oxide supported porous PtAu alloyed nanoflowers [19]. Very recently, we have reviewed the ultra-thin Pt NWs and their catalytic applications which were synthesized by acidic etching of FePt NWs [20]. In this chapter, we will summarize several Pt nanocomplexes that were produced on the basis of FePt NWs.

### 2.1. FePt@Cu NWs and the Catalytic Epoxidation

**Figure 1:**
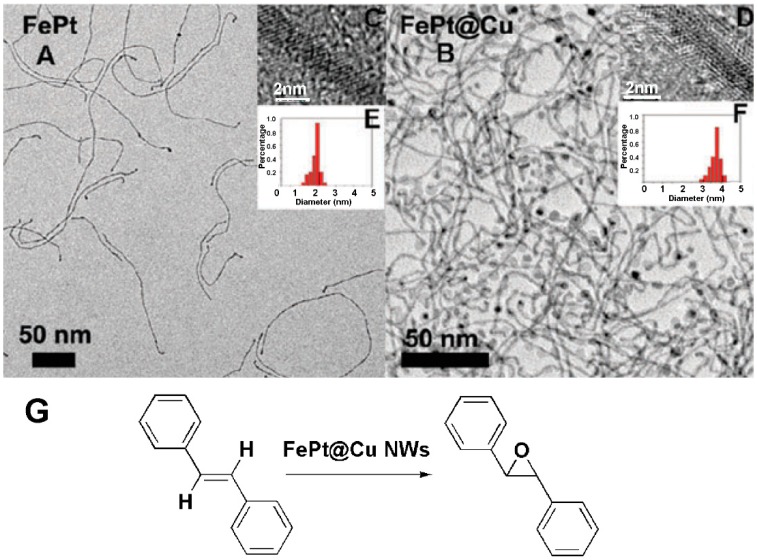
(**A**) The TEM and (**C**) high resolution TEM images of FePt nanowires (NWs); (**B**) The TEM and (**D**) high-resolution TEM images of FePt@Cu NWs; (**E**) The histograms of the diameters of FePt NWs and (**F**) FePt@Cu NWs; (**G**) The epoxidation of *trans*-stilbene using FePt@Cu NWs as catalysts [22].

Alkene oxidation is a key reaction in the organic reactions, and the corresponding products, including epoxides, aldehydes, ketones and carboxylic acids, are all essential precursors in the synthesis of various vital plasticizers, perfumes and epoxy resins [21]. In order to achieve these oxidations better, we have developed two novel kinds of Pt based NWs efficiently catalyzing alkenes, that is FePt@Cu NWs [22] and Pt@Fe_2_O_3_ NWs [23]. These NWs are introduced as follows. Firstly, by the reduction of cupric acetylacetonate on the surface of FePt NWs (Figure 1A,C,E) (Synthetic procedures: The mixed 200 mg Pt(acac)_2_ and 20 mL oleylamine were heated to 60 °C under N_2_ atmosphere to make it dissolve thoroughly. This solution was heated to 120 °C under stirring and then kept for 15 min. 150 μL Fe(CO)_5_ was injected into the hot solution and then the temperature was gradually raised to 160 °C. The reaction was kept at this temperature for 30 min without stirring. The black solution was then cooled to room temperature and centrifuged in excess ethanol.) [24], we prepared a novel FePt@Cu NWs (Figure 1B,D,F) that efficiently epoxidized stilbene in the presence of oxygen. The traditional production of epoxides shows non-selectivity and also leads to undesirable products resulting in chemical waste, as well as typically applied chemical oxidants like NaClO, PhIO and peracids [25,26,27]. However, these oxidants are often expensive and tend to be hazardous to handle. It is more favorable to use molecular oxygen as a green and environmentally friendly oxidant. Dating back to the 1980s, microporous titanium silicalite TS-1 as catalysts have been applied in alkene epoxidation (the oxidation of double bonds, which adds atomic oxygen in the two ends of atomic carbon, to form three-membered ring), yet it is unsuitable for the epoxidation of bulky alkenes. Given this context, novel metallic nanomaterials and molecular oxygen may be the most effective and suitable reaction media. Being similar to most of catalyses, a model catalytic reaction was first selected to investigate the activity of the catalysts. In the FePt@Cu NWs catalytic system, the epoxidation of *trans*-stilbene to form the corresponding product was conducted (Figure 1G). A series of different solvents, such as toluene, DMF, dioxane, methylcyclohexane and *o*-, *m*-, *p*-xylene were used to examine the solvent efficiency (the influence of different solvents on the efficiency of a certain reaction). The results show that it did not follow the solvent polarity, while aromatic hydrocarbons concluding with *p*-xylene and *o*-xylene were highly efficient solvents for the epoxidation. Moreover, in the two solvents, methylbenzaldehyde, tolylmethanol and methylbenzoic acid were obtained as byproducts. The resulting reaction mechanism was proposed that molecular oxygen was adsorbed on the surface of the catalysts firstly to form the active oxygen, then the solvent and substrate both reacted with the active oxygen. In detail, if the solvent reacted with the active oxygen, an unstable free radical intermediate was formed, which could then form peroxide in the oxygenated environment. Peroxide acted as an oxidant in the stilbene oxidation with the epoxy compound as the major product; if the solvent was more stable than stilbene, stilbene could react with the active oxygen and the epoxy compound was also the major product. On the other hand, temperatures of the catalytic system also affected the activity of catalysts. For example, when the temperature was below 60 °C, the catalytic reactions almost did not happen, while when the temperature was increased to 80 °C, the conversion was correspondingly increased to 9.8%; the most suitable temperature was 100 °C, and the yield of epoxidation product was 85.0%. It is worth noting that the FePt@Cu NWs could be recycled several times without any apparent catalytic degradation, the reason for which is mainly that the FePt@Cu nanostructure was retained as before and no significant loss of catalytic activity was observed which is similar to most metal nanocatalysts [28,29,30].

### 2.2. Pt@Fe_2_O_3_ NWs and the Selective Oxidation

**Figure 2:**
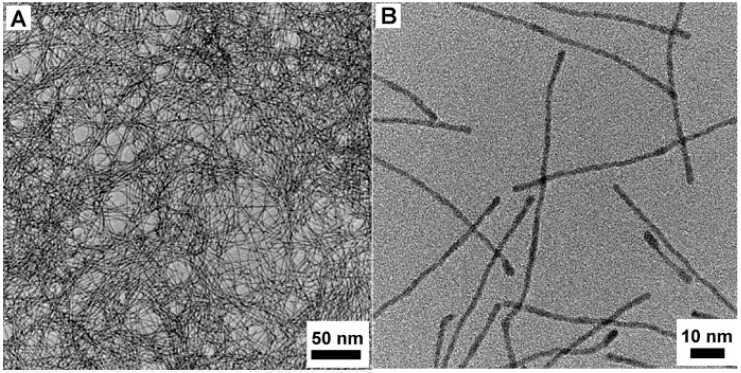

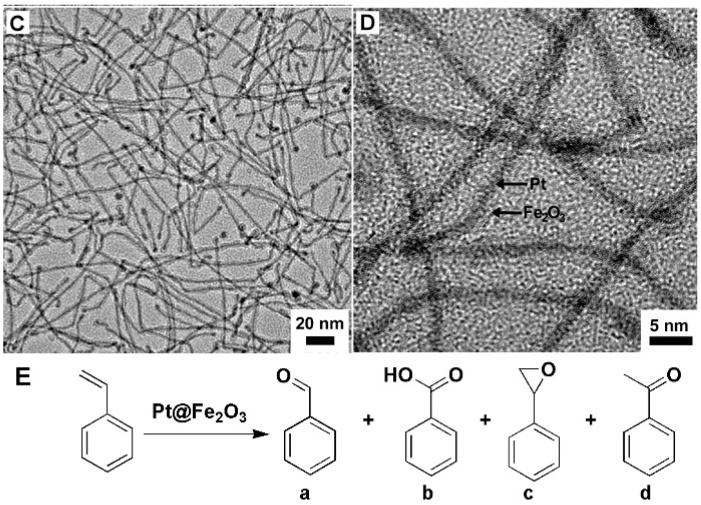
(**A**) The TEM and (**B**) high resolution TEM images of FePt NWs; (**C**) The TEM and (**D**) high resolution TEM images of Pt@Fe_2_O_3_ NWs; (**E**) The oxidation of styrene using Pt@Fe_2_O_3_ NWs as catalysts [23].

Secondly, Pt@Fe_2_O_3_ NWs (Figure 2C,D) were successfully fabricated by the oxygen oxidation of FePt NWs (Figure 2A,B) [24]. Similarly, this novel nanostructure can be used as heterogeneous catalysts in the selective oxidation of olefins and alcohols with high catalytic efficiency (Figure 2E). Although some other proposed metallic nanocatalysts have been developed for the selective oxidation [31,32,33,34], most of them failed to meet the green requirements (one of them is that catalysts must be able to be separated from target materials easily). Iron oxide, containing the low toxicity, mild conditions and high activity, has become a hot topic for green chemistry all over the world in recent years [35,36]. Meanwhile, one-dimensional (1D) nanostructures, which have a large surface area resulting in extremely higher catalytic activity than common nanocrystals catalysts in organic synthesis, have sparked an explosive interest among various promising nanomaterials investigated so far [37,38]. Thus, we attempted to synthesize a 1D iron oxide nanocatalyst to use for the catalytic oxidation. The resulting Pt@Fe_2_O_3_ NWs exactly exhibited a considerably better and excellent activity compared to previously-reported iron oxide nanoparticle catalysts. In order to show its high activity, different solvents, which are fairly significant for the reaction selectivity, were applied in the oxidation of styrene. Consistent with most of the literature demonstrated previously, acetonitrile is the most suitable solvent affording the highest yield of benzaldehyde [39,40,41,42]. The reaction temperature and oxygen pressure are both important factors. With the temperature and oxygen pressure increasing, although the styrene conversion increased, the selectivity of benzaldehyde decreased. Like other 1D nanocatalysts, this catalyst can also be recycled by simple centrifugation [29,30,43]. Slightly different from others, the conversion and the yield of second run are lower than that of the first run, which denotes that the catalytic activity of fresh catalyst is better than that of the recycled catalyst. The most important factor is the small quantity of benzoic acid generated in the first reaction which etched the Fe_2_O_3_ and partially deactivated the Fe_2_O_3_. The common applicability of this NW catalyst was investigated with a variety of substituted styrene compounds, the results of which showed that the electron-donating substituents afforded a higher yield indicating that the substituent had a significant influence on the yield of the products [44,45,46]. In addition, with Pt@Fe_2_O_3_ NWs as the catalysts, alcohols can also be smoothly converted to the corresponding aldehydes or ketones.

### 2.3. Bimetallic Pt@Ir Nanocomplexes and the Catalytic Hydrogenation

**Figure 3:**
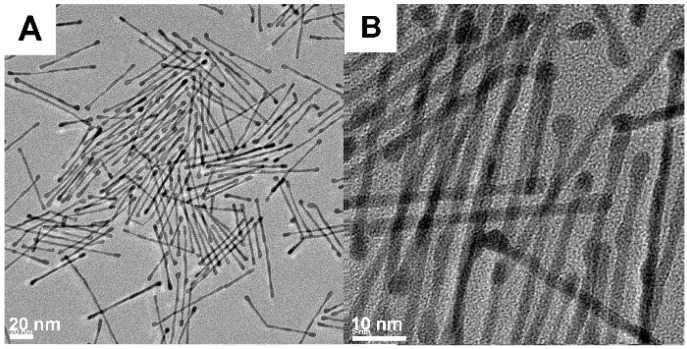

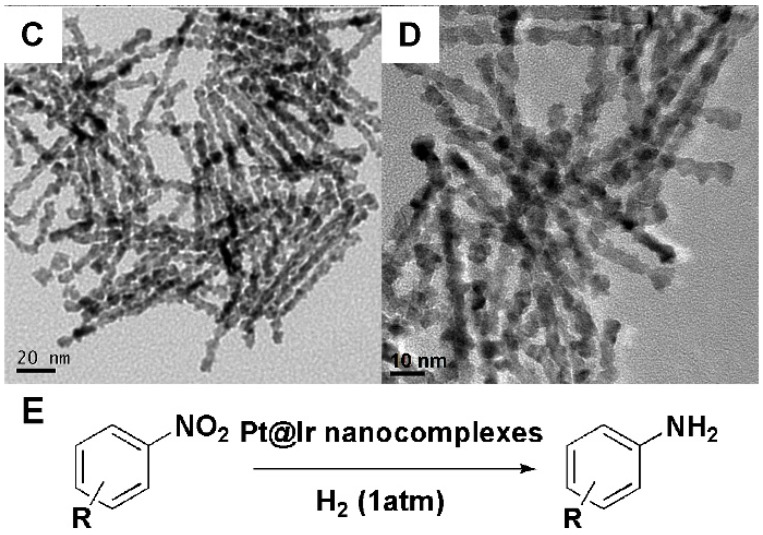
(**A**) The TEM and (**B**) high resolution TEM images of FePt nanorods; (**C**) The TEM and (**D**) high resolution TEM images of Pt@Ir nanocomplexes; (**E**) The hydrogenation of aromatic nitrobenzene using Pt@Ir nanocomplexes as catalysts [47].

Lastly, a bimetallic Pt@Ir zigzag nanocomplex was successfully prepared (Figure 3A–D). Using this novel nanocomplex as the catalyst, a high catalytic activity was observed in hydrogenations under mild conditions[47]. In this synthetic process, Pt@Ir nanocomplexes were produced by the preparation of Pt nanorods [24] and subsequent growth of Ir on the Pt nanorods. Furthermore, this morphology has not been reported in nanostructures with the same chemical composition before our group’s work. Using the Pt@Ir nanocomplexes as catalysts, a series of amine compounds were successfully synthesized (Figure 3E), which are important intermediates in the industrial production of various pharmaceuticals, dyes, agrochemicals and polymers [48,49,50].

We have previously studied Pt NWs in a series of organic hydrogenations and obtained outstanding results [51,52,53]. Irrespective of hydrogenations, a number of oxidations have been demonstrated by using FePt@Cu NWs and Pt@Fe_2_O_3_ NWs synthesized based on FePt NWs. Inspired by these results, we discuss whether other metals (such as Au and Pd) can be composited with Pt nanostructures to form various novel nanocomplexes that can be used as catalysts with high activities for electro-catalysis, PtAu and PtPd nanocomplexes in the next chapter.

## 3. Bimetallic Pt-M (Au and Pd) Nanocomplexes and Their Electro-Catalysis

In addition to organic catalysis, electro-catalysis is another focus of contemporary catalytic reactions [54,55,56]. A direct methanol fuel cell is one of the most ideal mobile energy forms, which is a kind of proton exchange membrane fuel cell with high energy conversion efficiency, a simple structure and environmentally friendly properties [57,58,59]. Pt is the most effective electro-catalyst. However, its catalytic performance cannot meet the practical requirements. On the one hand, the amount of Pt used on the Pt electrode in the oxidation of organic small-molecules is quite large. On the other hand, the Pt catalyst is easily poisoned by toxic intermediates (CO) during oxidation. In view of fuel cells, in order to decrease the amount of noble metallic catalysts as well as to increase their catalytic activity (a property of the catalyst under certain conditions, in relation to a specific chemical reaction), we must improve the dispersion of noble metals in the matrix. Furthermore, it is necessary to introduce other metallic components that cooperate with Pt to reduce the oxidation overpotential and catalyst poisoning of organic small-molecules, so as to improve the performance of the catalysts comprehensively. Thus, hybrid bimetallic nanomaterials exhibit better electro-catalytic activity due to the incorporation of two distinct nanostructures into a single material [60,61,62]. Many works focus on PtRu bimetallic nanomaterials which are the most effective electrocatalysts currently [11,12,13,14,15]. In 2003, Xin’s group reported that the presence of Sn, Ru and W enhanced the activity of Pt towards ethanol electro-oxidation [11]. Recently, Xing and coworkers synthesized a highly active PtRu alloy nanosponge for methanol electro-oxidation which greatly increased Pt utilization and anti-CO poisoning ability [15].

In this review, the PtPd bimetallic nanocomplex and two kinds of PtAu bimetallic nanocomplexes are reviewed as electro-catalysts for the catalytic reaction. Besides excellent catalytic activities, these bimetallic nanocatalysts partly increased the Pt utilization and anti-CO poisoning ability. One of the most effective approaches for synthesizing hybrid nanostructures with the controlled morphology and composition is to use a simple unit as the building block, followed by nucleation and growth [63,64,65,66].

### 3.1. Pt-Au Heterostructures and the Oxygen-Reduction Reaction

**Figure 4:**
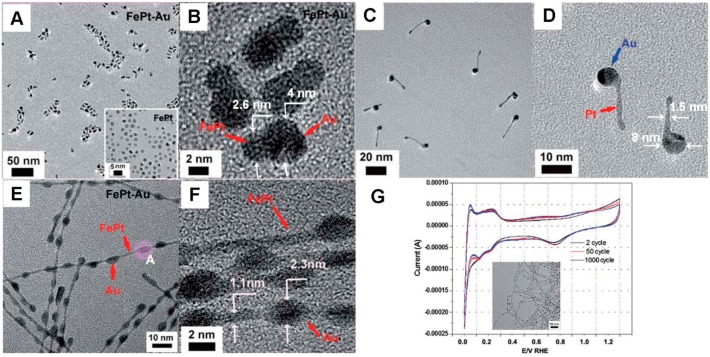
(**A**) The TEM and (**B**) high-resolution TEM image of PtAu heterodimers; (**C**) The TEM and (**D**) high-resolution TEM image of PtAu tadpole-like hybrid structures; (**E**) The TEM and (**F**) high-resolution TEM image of PtAu necklace-like hybrid structures; (**G**) The oxygen-reduction reaction using PtAu necklace-like hybrid structures as electrocatalysts [67].

In our group, we firstly described the selective growth of Au nanoparticles onto a series of nanomaterials (nanoparticles, nanorods and nanowires) to form heterodimers (Figure 4A,B), tadpole-like (Figure 4C,D) and necklace-like (Figure 4E,F) hybrid structures [67]. In these processes, Au occurred more rapidly in areas with high chemical potentials in the nucleation and growth procedures, and the nanorod’s length might have an influence on the growth and formation of hybrid nanostructures. Another key factor, that plays a very important role in the heterostructure control of hybrid nanomaterials, is the added precursors followed by the first nano-units. For example, when adding Au^+^ to the solution of Pt NWs, a necklace-like Pt-Au heterostructure was successfully synthesized; while when Au^3+^ precursors were added, Au nanoparticles grew at the tip of Pt NWs, because that Au^+^ has a higher activity and more easily forms Au^0^ compared to Au^3+^. On the basis of this controlled synthesis, the necklace-like Pt-Au heterostructure was selected as the electrocatalyst for the oxygen-reduction reaction by using voltammetry (Figure 4G). Measuring H adsorption before and after potential cycling can effectively determine the Pt surface area of the Pt-Au electrode. Furthermore, this electro-catalytic system showed no obvious change after 50 and 1000 cycles, indicating little recordable loss of the Pt surface area.

### 3.2. Au-Pt Bimetallic Nanocomplexes and the Electro-Catalysis

**Figure 5:**
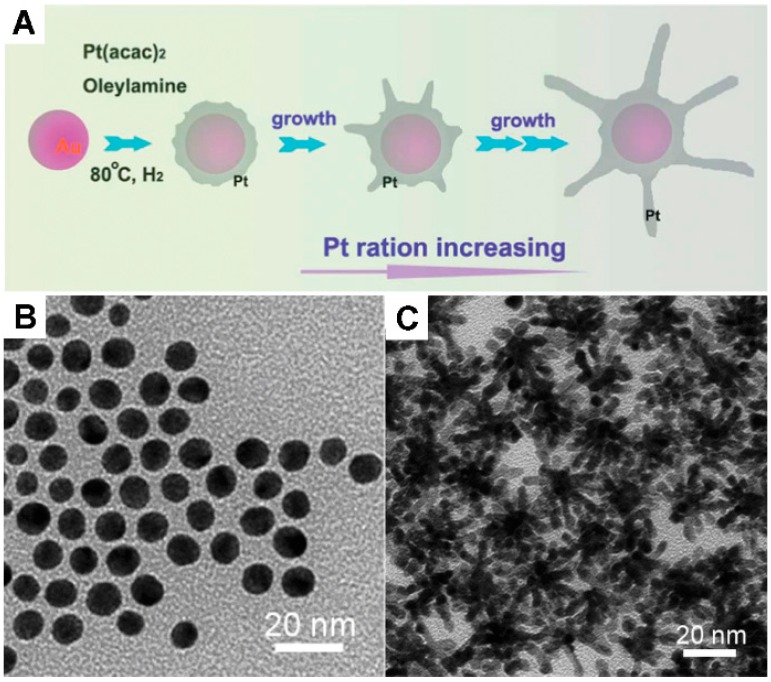
(**A**) The synthetic scheme of Au-Pt bimetallic nanocomplexes; (**B**) The TEM image of Au nanoparticles; (**C**) The TEM image of Au-Pt bimetallic nanocomplexes [68].

By using the same two-steps, the seed-growth method, another high-quality 3D bimetallic nanostructure has also been successfully prepared, that is Au-Pt bimetallic nanocomplexes (Figure 5C) [68]. In the synthesis of this bimetallic nanostructure, oleylamine was applied as a reducing agent and hydrogen was used to control the morphology. Firstly, Au nanoparticles with a diameter of 8 nm were prepared and dispersed in oleylamine for further use (Figure 5B), followed by an appropriate amount of Pt(acac)_2_ added and reduced on the surface of Au seeds under 1 bar of H_2_ atmosphere. The usage of hydrogen is very vital in the growth of Pt nanobranches, in which Pt(acac)_2_ was reduced forming nanocrystals on the surface of Au seeds, and the partially hydrogen-active surfaces of Pt nanocrystals were covered by H_2_ inhibiting the crystal growth that resulted in Pt nanocrystals to form metallic branches. The electrocatalytic activity of this Au-Pt bimetallic nanocomplex was examined via direct oxidation of methanol and formic acid and exhibited enhanced catalytic activity to a great extent. For the methanol oxidation, the mass-normalized current density of the Au-Pt bimetallic nanocomplexes (371.8 mA·mg^−1^ Pt) was 1.44-times larger than that of the commercial catalysts (JM-Pt/C, 257.4 mA·mg^−1^ Pt) which showed a great increase in mass current density. For the formic acid oxidation, the mass-normalized current density of Au-Pt bimetallic nanocomplexes (137.1 mA·mg^−1^ Pt) was 1.41 times larger than that of commercial catalysts (JM-Pt/C, 97.5 mA·mg^−1^ Pt) which also showed a great increase in mass current density.

### 3.3. Pt/Pd Bimetallic Nanodendrites and the Electro-Catalysis

**Figure 6:**
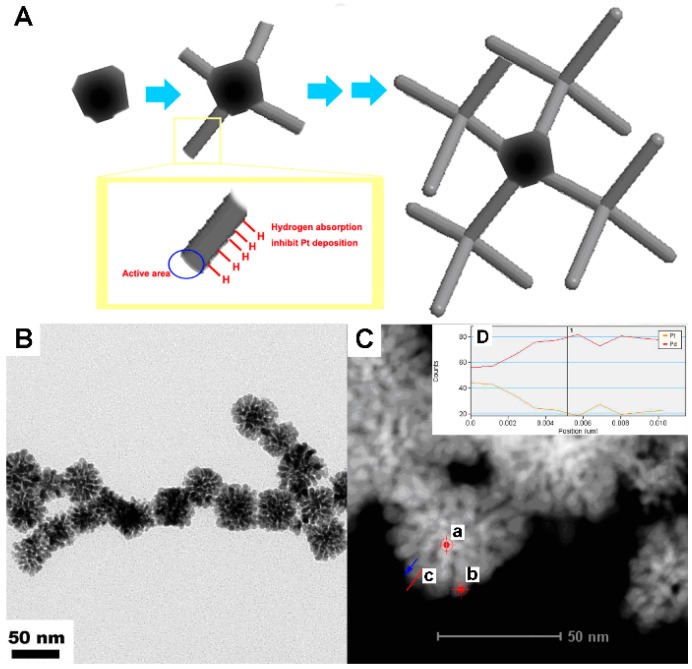
(**A**) The synthetic scheme of Pt/Pd bimetallic nanodendrites; (**B**) The TEM image of Pt/Pd bimetallic nanodendrites; (**C**) The STEM image of Pt/Pd bimetallic nanodendrites as catalysts; (**D**) The line spectrum of c. spot a: both Pt and Pd elements, spot b: only Pd element) [69].

Another synthetic approach for bimetallic nanostructures is the one-pot wet chemical method. Two precursors of different metals are added in the reaction simultaneously and the mole ratio of two metals can be controlled easily by regulating the ratio of two precursors, which offers a facile and efficient route for preparing nanodimensional structures. For example, our group reported 3D Pt/Pd bimetallic nanodendrites (Figure 6) using oleylamine as the reducing agent and H_2_ for controlling the morphology [69]. Being similar to the synthesis of Au-Pt nanocomplexes [68], H_2_ is essential to this synthetic reaction as a capping agent and partially capped hydrogen-active surface, which resulted in the second generation of metallic branches on the tips of active branches. Compared to Pt nanoparticles [70], this Pt/Pd bimetallic nanodendrite showed much better and excellent electrocatalytic activity for the oxidation of methanol.

## 4. Au, Pd, Ag Nanostructures and Catalytic Applications in Organic Reactions

Pt-based nanomaterials have been widely used in catalysis due to their superior chemical stability and catalytic activity. In addition, other noble metallic nanomaterials including Au, Pd, Ag *etc.* have also been attracting much attention in a variety of fields, especially as catalysts in organic reactions. 

### 4.1. Au NWs and the Selective Oxidation

**Figure 7:**
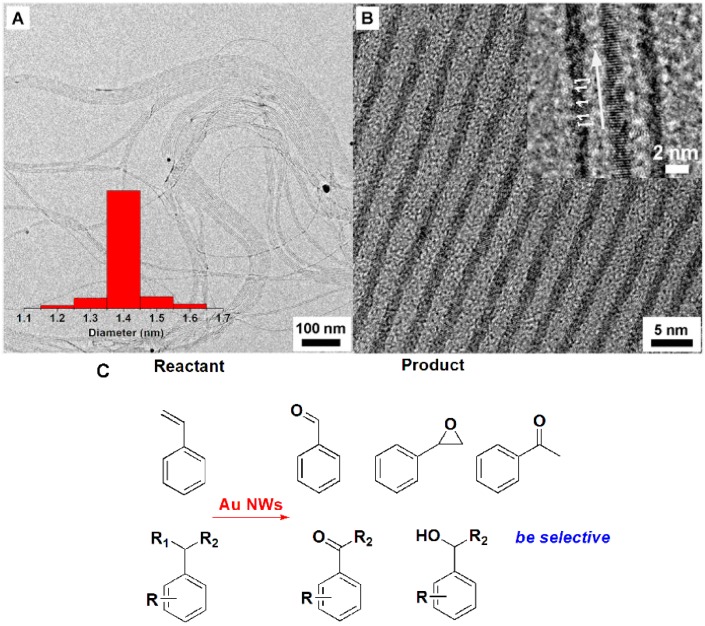
(**A**) The TEM and (**B**) high resolution TEM images of Au NWs; (**C**) The oxidation of styrene and ethylbenzene using Au NWs as catalysts [80].

Dating back to 1991, Hutchings and coworkers developed Au nanocatalysts for the hydrochlorination of acetylene [71], which officially opened the prelude of nano-Au-catalyzed reactions. Additionally, more and more researchers have been joining in the area of Au nanocatalysis. Au nanomaterials are one of the most important catalysts for oxidation [72,73] and play a key role in the epoxidation of propylene. However, all of the Au catalysts with catalytic activity for propylene have to be loaded on titanium supports [74]. For example, Caps and coworkers demonstrated that Au/TiO_2_ as catalysts showed quite high catalytic activity for the epoxidation of *cis*-stilbene [75,76,77,78,79]. Irrespective of that, the low catalytic activity of Au catalysts also inhibited their applications in industry. Here, we review an improved methodology for the synthesis of Au NWs (Figure 7A,B) and their catalytic activity for styrene, as well as ethylbenzene oxidation(Figure 7C) [80]. Following a reported method [81], Au NWs with an average diameter of 1.4 nm and several micrometers in length have been prepared after some minor modification. The oxidation of styrene and alkylbenzene were both studied to evaluate the Au NWs’ activity. Table 1 and Table 2 show the catalytic performance of Au NWs on the oxidation of styrene and alkylbenzene, respectively.

**Table 1:** Catalytic performance of Au NWs on the oxidation of styrene.

Entry	Solvent	Conversion (%)	Selectivity (%)		
			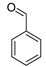	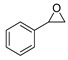	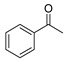
1	DMF	23.2	27.9	63.3	8.8
2	1,4-dioxane	81.7	38.5	53.9	1.6
3	*m*-xylene	22.7	70.5	19.2	10.3
4	*p*-xylene	39.5	62.5	23.8	13.7
5	chlorobenzene	3.9	69.3	30.7	-
6	heptane	28.6	50.6	5.7	3.8
7	toluene	17.0	87.2	9.9	0.9

All reactions were carried out with 1.25 g styrene and examined by GC-MS.

**Table 2:** Catalytic performance of Au NWs on the oxidation of alkylbenzene.

Entry	R	R_1_	R_2_	Conversion (%)	Selectivity (%)	
					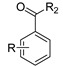	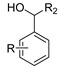
1	H	H	H	1.3	95.2	4.8
2	*o*-CH_3_	H	H	4.3	62.1	37.9
3	*m*-CH_3_	H	H	1.3	100	-
4	*p*-CH_3_	H	H	3.4	82.4	17.6
5	*p*-Cl	H	H	1.0	100	-
6	*p*-OCH_3_	H	H	0.6	100	-
7	H	H	CH_3_	21.1	100	-
8	*p*-NO_2_	H	CH_3_	23.1	83.5	16.5
9	*p*-OCH_3_	H	CH_3_	1.3	70.8	29.2
10	H	CH_3_	CH_3_	47.9	62.9	37.1

All reactions were carried out with 1.25 g alkylbenzene and examined by GC-MS.

### 4.2. CuO@Ag NWs and the Selective Oxidation

**Figure 8:**
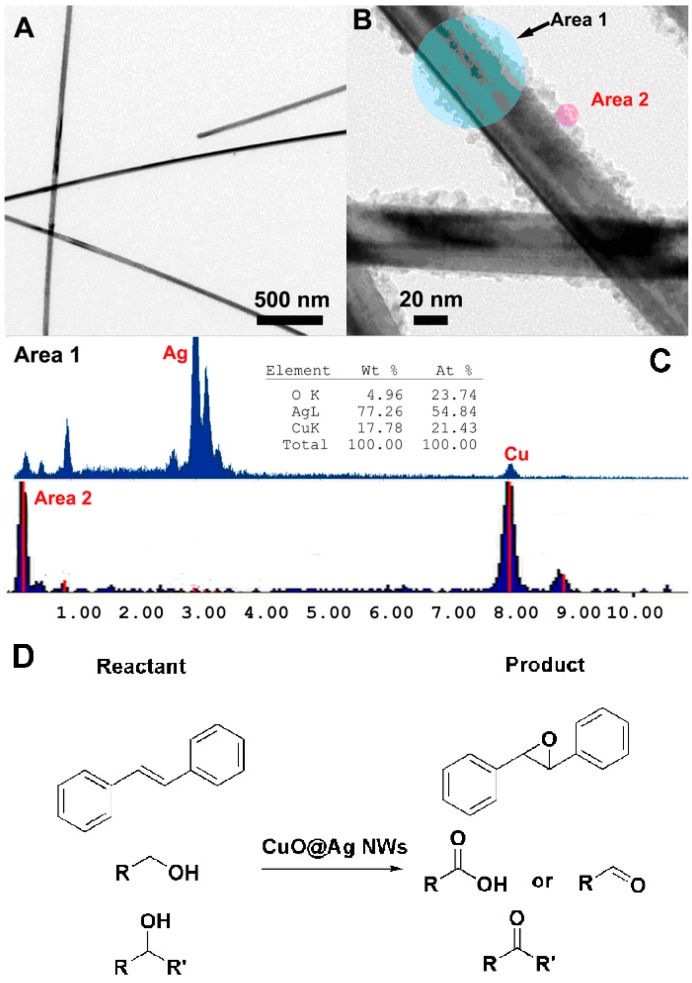
(**A**) The TEM image of Ag NWs; (**B**) The TEM image of CuO@Ag NWs; (**C**) The EDS spectrum of CuO@Ag NWs; (**D**) The epoxidation of *trans*-stilbene and the oxidation of alcohols using CuO@Ag NWs as catalysts [86].

In recent years, that the preparation of Ag NWs has been successfully industrialized, resulting in the various syntheses of Ag wires-based nanocomplexes. Xia’s group reported a series of Ag nanostructured materials [82,83,84] and reviewed several synthetic approaches including the seed-directed growth for Ag NWs [85]. Herein, we review related developments in our group regarding Ag NWs carrying CuO nanoparticles [86]. The nanomaterials (CuO@Ag NWs) were synthesized via a simple route which consisted of Ag NWs and subsequent deposition of CuO nanoparticles on them (Figure 8). The common metal copper was introduced in the noble metal Ag, which showed excellent catalytic activity as well as improved economic benefits [87,88,89,90,91]. With CuO@Ag NWs as catalysts, the epoxidation of *trans*-stilbene was carried out under the green oxidant air with outstanding activity and selectivity (Figure 8D). Furthermore, the CuO@Ag NWs could also be recycled without a significant loss in activity which also met the green requirements. Furthermore, these nanowires also exhibited good catalytic activity in the oxidation of alcohols with *tert*-butyl hydroperoxide as oxidants (Figure 8D). At the same time, Ag NWs and free CuO nanoparticles were performed as catalysts under the same reaction conditions. Using Ag NWs as catalysts, only a 40% conversion with a selectivity of 95% could be detected. Although the corresponding product from *trans*-stilbene could be achieved in a yield of 80% in the first use of free CuO nanoparticles, they could not be recycled for further use due to their aggregation [92]. The corresponding results proved that CuO@Ag NWs showed much higher activity and stability than Ag NWs and free CuO nanoparticles. These demonstrations may help with the potential applications of common metal combined with noble metals instead of single noble metals.

### 4.3. Pd Nanocatalysts and the Reductive Coupling Reactions

**Figure 9:**
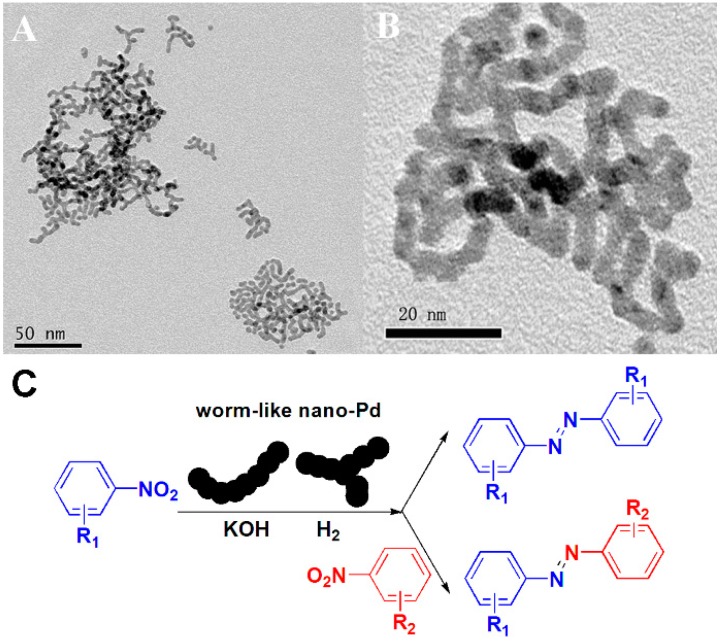
(**A**) The TEM image of worm-like Pd nanostructures; (**B**) The high resolution TEM image of worm-like Pd nanostructures; (**C**) The formation of aromatic azo compounds using worm-like Pd nanostructures as catalysts [93].

**Figure 10:**
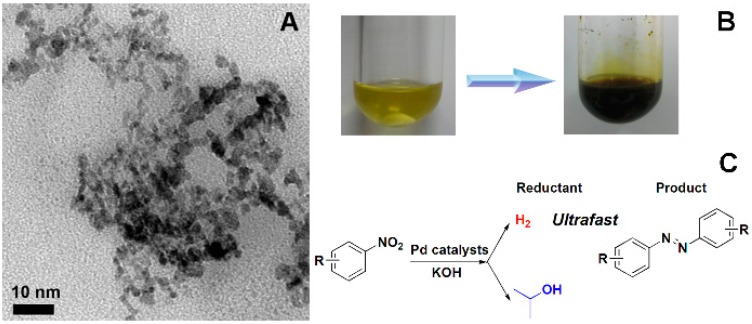
(**A**) The TEM image of Pd nanoclusters generated *in situ*; (**B**) The optical image of reactions before and after the formation of aromatic azo compounds; (**C**) The formation of aromatic azo compounds using Pd nanoclusters generated *in situ* as catalysts [94,95].

Meanwhile, our group has investigated a series of nano Pd catalysts for catalysis such as worm-like nano Pd [93] and Pd nanoclusters generated *in situ* [94,95]. Both of these showed high activity towards the synthesis of aromatic azo compounds. Aromatic azo compounds are high-value materials and widely used in the chemical industry as indicators [96,97], drugs [98,99], organic dyes [100] and food additives [101,102]. Palladium is one of the most useful catalysts both in hydrogenation and coupling reactions. Many works have reported that the shape of nanomaterials affected the catalytic activity [103,104]. In 2007, Kim and coworkers provided the first report by using a Pd nanocatalyst, which was prepared as Pd nanoparticles entrapped in aluminum hydroxide, for amine racemization [105]. Huang’s group demonstrated another extremely active supported Pd nanocatalyst for the Suzuki-Miyaura reaction [106]. Very recently, Li and coworkers presented the direct synthesis of hybrid layered double hydroxide-carbon composite supported Pd nanocatalysts for the selective hydrogenation of citral efficiently [107]. Besides these supported nanomaterials, unsupported Pd nanostructures have also been investigated such as the magnetic Pd/C@Fe_3_O_4_ spheres reported by Zhang’s group [108]. Not just in organic catalysis, Pd nanomaterials have also been widely used in electro-catalysis. Hierarchical flower-like ZnO microspheres composed of porous nanosheets were used as a new support for a Pd catalyst, which exhibited excellent catalytic activity for CO oxidative coupling to dimethyl oxalate [109]. In the last chapter, we reviewed the Pt/Pd bimetallic nanodendrites and their electro-catalysis. Here, we review an improved methodology for the synthesis of worm-like Pd catalysts and a kind of Pd nanoclusters generated *in situ* for organic catalysis in our group. In the synthesis of this worm-like Pd, palladium diacetate, *n*-hexadecyl trimethyl ammonium bromide and 1-dodecylamine were dissolved in toluene. The dropwise addition of fresh NaBH_4_ solution was carried out as the reductant and followed by the mixture being stirred for 1 h at room temperature. The diameter of this obtained worm-like Pd was ~3.5 nm (Figure 9A,B). Using this novel catalyst, the formation of aromatic azo compounds is efficient, and does not need any harmful reagents which makes the synthetic approach more environmentally friendly (Figure 9C). Later, our group also developed an efficient catalytic system based on Pd nanoclusters generated *in situ* for the synthesis of aromatic azo compounds (Figure 10). In this catalytic system, we successfully combined the nanomaterial synthesis and aromatic azo compounds in a one-pot reaction. Furthermore, the *in situ*-prepared Pd nanoclusters exhibited higher catalytic activity than other Pd nanocatalysts synthesized in advance. This investigation may provide new methodology and thinking for nanomaterial synthesis and catalytic applications.

## 5. Perspectives and Challenges

With the continuous development of nanomaterials’ synthetic technology and the expansion of their application scopes, the industrial production of nanomaterials will have a great impact on the traditional chemical industry and its related industries. Metallic nanomaterials, a new type of nanomaterial, have tantalizing prospects in the fields of microelectronics, optoelectronics, and sensors, especially in catalytic areas, due to the excellent electrical, optical, magnetic and thermal properties. However, there are only a few of methods for the preparation of metallic nanomaterials, and it is quite difficult to achieve the industrialization of developed products. Therefore, there is still a long way to realize the industrial application of metallic nanomaterials. Furthermore, there are several problems to be solved. The first problem in the industrialization is how to solve the mass production of metallic nanomaterials. Secondly, as catalytic materials, it is very vital to construct novel structures and compositions on the nanometer scale, so as to design and synthesize appropriate catalysts used in various reactions. By investigating the influence of a nanomaterial’s crystal surface, morphology and metallic valence state on the catalytic activity, the key factors of a nano-catalytic system are further studied to discuss catalytic mechanisms. Under green reaction conditions, it is also quite important to maintain the high activity of catalysts that is how to avoid poisoning for solving the recyclability of catalysts. Thirdly, for economic efficiency, developing some inexpensive non-noble metallic nanocrystals for the substitution of expensive noble metallic nanomaterials is another severe challenge. Lastly, because the application scopes of metallic nanomaterials are still relatively narrow, it also becomes very significant to explore the applications of metallic nanomaterials actively in other fields and broaden the breadth and sophistication of their applications in materials science, chemistry, biology, physics and some interdisciplines.
